# Mindfulness and mental health: Dispositional mindfulness, psychopathology, and subjective well-being in a Portuguese community sample

**DOI:** 10.1192/j.eurpsy.2026.10770

**Published:** 2026-07-31

**Authors:** L. Lemos, I. Pires, D. Andrade, F. Nogueira, D. Azedo, F. Daniel, H. Espírito-Santo

**Affiliations:** 1 Instituto Superior Miguel Torga; 2 Centro de Inovação em Biomedicina e Biotecnologia, Universidade de Coimbra, Coimbra, Portugal

## Abstract

**Introduction:**

Dispositional mindfulness has been associated with better mental health and well-being, yet its pathways in community samples remain under-specified.

**Objectives:**

To examine (i) age/sex differences in mindfulness, psychopathology, and well-being; (ii) correlations among dispositional mindfulness, symptom severity, and subjective well-being; and (iii) whether mindfulness mediates links between psychopathology and well-being.

**Methods:**

Cross-sectional online survey with a non-probabilistic snowball sample of Portuguese adults (N = 400; 18–78 years). Measures: Mindfulness Attention Awareness Scale (MAAS), Brief Symptom Inventory-18 (global severity index and subscales), Positive and Negative Affect Schedule (PANAS), and Satisfaction With Life Scale (SWLS). Analyses included t-tests/ANOVA, Pearson correlations, and mediation models with MAAS as mediator.

**Results:**

Younger adults showed higher psychopathology and negative affect, whereas adults aged 60–69 reported higher mindfulness and life satisfaction (declining ≥70 years). Men scored higher on mindfulness; women reported higher negative affect.

Mindfulness correlated positively with life satisfaction and positive affect, and negatively with symptom severity (IGG; somatization, depression, anxiety), stress, and negative affect.

In mediation analyses, psychopathology had a direct negative effect on life satisfaction and a direct positive effect on negative affect; the direct path to positive affect was not significant. Total effects were negative for life satisfaction and positive for negative affect. Models specified MAAS as mediator between psychopathology and well-being indicators.

**Conclusions:**

Findings indicate that higher dispositional mindfulness is linked to lower symptom burden and better subjective well-being, partially accounting for the association between psychopathology and well-being. Results support integrating mindfulness-based strategies in mental-health promotion for adults. Limitations include cross-sectional design, online snowball sampling, over-representation of women/younger adults, and reliance on self-report.

**Disclosure of Interest:**

None Declared

